# The Theory of Planned Behavior: Selected Recent Advances and Applications

**DOI:** 10.5964/ejop.v16i3.3107

**Published:** 2020-08-31

**Authors:** Michael Bosnjak, Icek Ajzen, Peter Schmidt

**Affiliations:** a ZPID – Leibniz Institute for Psychology Information, Trier, Germany; b Department of Psychological and Brain Sciences, University of Massachusetts Amherst, Amherst, MA, USA; c Centre for Development and Environment (ZEU), University of Gießen, Gießen, Germany; d Department of Psychosomatic Medicine, University of Mainz, Mainz, Germany

**Keywords:** editorial, theory of planned behavior, theory adjustments, theory extensions, theory applications

## Abstract

This editorial gives a brief introduction to the articles included in the thematic section of Europe's Journal of Psychology, which is devoted to selected recent advances and applications of the theory of planned behavior (TPB). The five contributions address two thematic streams: (1) adjustments and extensions of the original theory and (2) applications of the TPB in public health and the political sciences.

Psychology must be practiced by nonpsychologists. … the secrets of our trade need not be reserved for highly trained specialists. Psychological facts should be passed out freely to all who need and can use them. … There simply are not enough psychologists, even including nonprofessionals, to meet every need for psychological services. The people at large will have to be their own psychologists, and make their own applications of the principles that we establish. … Our responsibility is less to assume the role of experts and try to apply psychology ourselves than to give it away to the people who really need it. (Miller, 1969, pp. 1070-1071)

— George Miller, “giving psychology away” presidential address to the American Psychological Association 1969.

As of April 2020, the theory of planned behavior (TPB; Ajzen, 1991, 2012) has been subject to empirical scrutiny in more than 4,200 papers referenced in the Web of Science bibliographic database, rendering it one of the most applied theories in the social and behavioral sciences. A thematic treemap analysis (Figure 1) reveals that the TPB has received broad attention in areas such as the health sciences, environmental science, business and management, and educational research, fulfilling George Miller´s “giving psychology away” request in an ideal sense.

**Figure 1:**
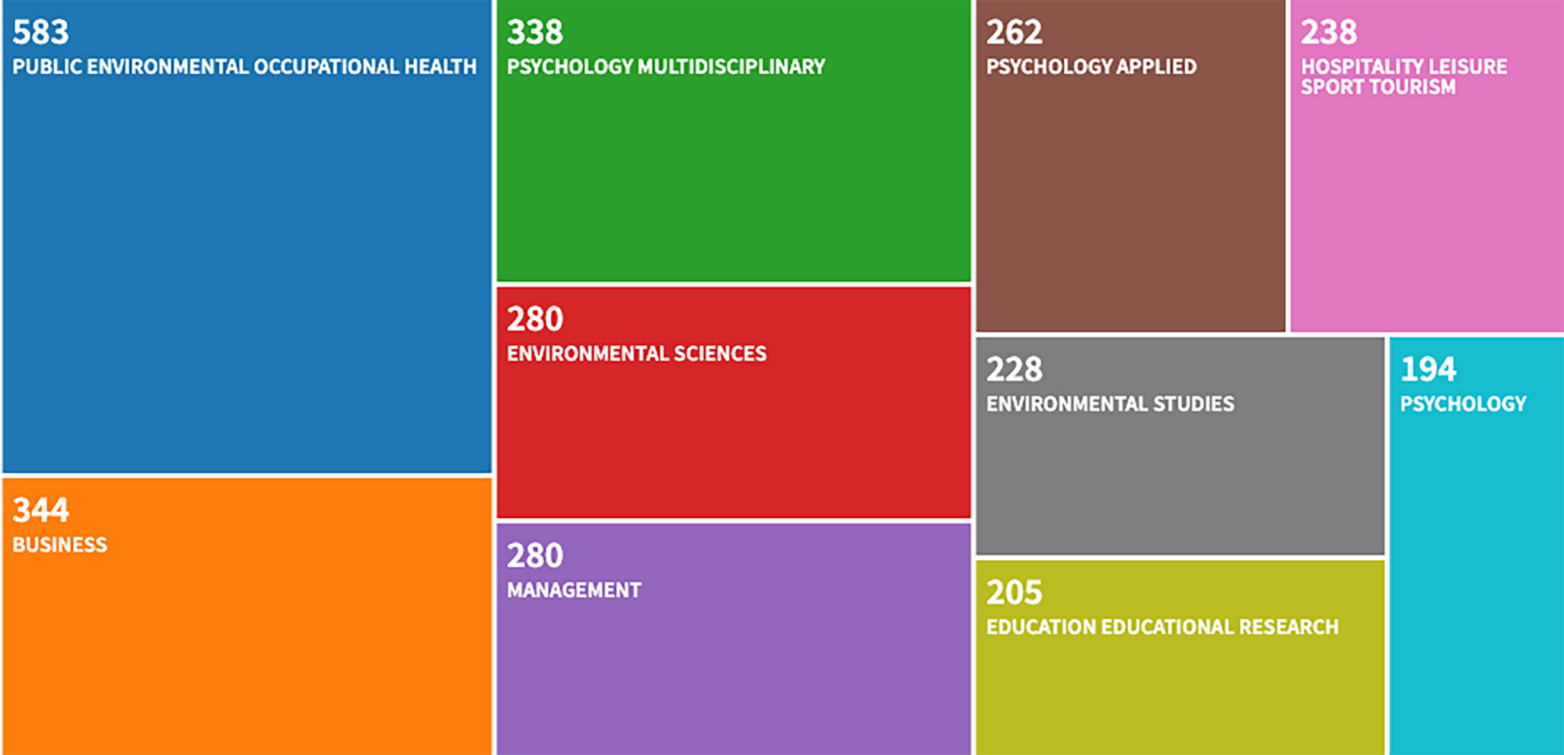
Treemap of the behavioral domains in which the theory of planned behavior has been used according to the Web of Science bibliographic database (as of April 2020). 2952 out of 4230 manuscripts could be assigned to the ten most covered thematic areas.

According to the TPB, human behavior is guided by three kinds of considerations: beliefs about the likely consequences of the behavior (*behavioral beliefs*), beliefs about the normative expectations of others (*normative beliefs*), and beliefs about the presence of factors that may facilitate or impede performance of the behavior (*control beliefs*). In their respective aggregates, behavioral beliefs produce a favorable or unfavorable attitude toward the behavior; normative beliefs result in perceived social pressure or subjective norm; and control beliefs give rise to perceived behavioral control or self-efficacy. The effects of attitude toward the behavior and subjective norm on intention are moderated by perception of behavioral control. As a general rule, the more favorable the attitude and subjective norm, and the greater the perceived control, the stronger should be the person’s intention to perform the behavior in question. Finally, given a sufficient degree of actual control over the behavior, people are expected to carry out their intentions when the opportunity arises. Intention is thus assumed to be the immediate antecedent of behavior. To the extent that perceived behavioral control is veridical, it can serve as a proxy for actual control and contribute to the prediction of the behavior in question (Ajzen, 2019a). Figure 2 is a schematic representation of the theory.

**Figure 2:**
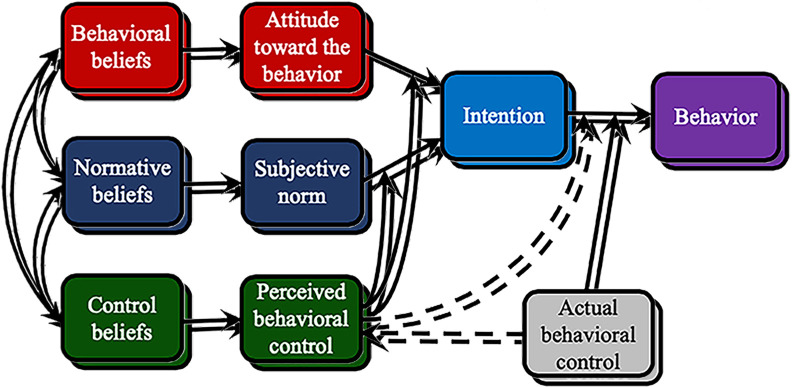
Graphical depiction of the theory of planned behavior (Ajzen, 2019b).

The goal of this editorial is to give a brief overview of the articles in this thematic section of Europe's Journal of Psychology, which is devoted to selected recent advances and applications of the TPB. The five contributions address two thematic streams: (1) adjustments and extensions of the original theory and (2) applications of the TPB in public health and the political sciences.

## Adjustments and Extensions

In a contribution entitled “The theory of planned behavior and the social identity approach: A new look at group processes and social norms in the context of student binge drinking”, Loren Willis, Eunro Lee, Katherine Reynolds, and Kathleen Klik explore whether social identity acts as a driver of existing TPB constructs and may help to explain how abstract group processes impact student binge drinking behavior (Willis, Lee, Reynolds, & Klik, 2020). Specifically, the interaction between group identification and the importance of drinking to the group’s identity significantly predicted an individual’s attitudes towards binge drinking and perceived social binge drinking norms (descriptive and injunctive), which in turn predicted intentions to binge drink.

Luigina Canova and Anna Maria Manganelli contributed a paper entitled “Energy-saving behaviours in workplaces: Application of an extended model of the theory of planned behaviour” (Canova & Manganelli, 2020). The aim of their study was to explore the determinants of two specific energy-saving behaviors, namely ‘switching off non-essential lights’ and ‘completely switching off electronic devices’. An extended TPB model was employed, considering two components (affective and cognitive) of the attitude towards these behaviours and then adding habit as a new variable. A two-wave prospective study that assessed the presumed antecedents of energy-saving behaviour (Wave 1) and the self-reported behavior one month later (Wave 2) showed that the inclusion of habit improved the predictive power of the TPB. Cognitive attitude, subjective norm, perceived behavioural control, and habit were significantly related to intentions, and perceived behavioural control was the strongest predictor. In addition, habit moderated some relationships between the TPB constructs and intentions.

A different aspect of the TPB is addressed in an article by Francesco La Barbera and Icek Ajzen entitled “Control interactions in the theory of planned behavior: Re-thinking the role of subjective norm” (La Barbera & Ajzen, 2020a). The authors shed light on perceived behavioral control (PBC) as a moderator of attitude (ATT) and subjective norm (SN). In three studies dealing with different behaviors (voting, reducing household waste, and energy consumption) the authors show that greater PBC tends to strengthen the relative importance of ATT in the prediction of intention, whereas strong PBC tends to weaken the relative importance of SN. The latter pattern was observed in relation to injunctive as well as descriptive subjective norms, and it may help explain the relatively weak direct relation between SN and INT frequently observed in TPB studies.

## Applications in Public Health and Political Science

The paper contributed by Andrea Caputo (“Comparing theoretical models for the understanding of health-risk behavior: Towards an integrative model of adolescent alcohol consumption”) applied the TPB in combination with the prototype-willingness model (PWM) to predict risky alcohol consumption among adolescents (Caputo, 2020). In essence, PWM considers prototype favourability and similarity as an additional behavioral antecedent. The findings show that attitudes and subjective norms served as the best predictors. However, the integrative model combining TPB and PWM had greater explanatory power and provided a better fit to the data compared to any single model. Overall, the perceived social approval from significant others and the volitional component seemed to play a central role in understanding adolescents’ alcohol consumption.

The fifth paper in this thematic section by Francesco La Barbera and Icek Ajzen entitled “Understanding support for European integration across generations: A study guided by the theory of planned behavior” explores the antecedents of voting for EU integration in an Italian convenience sample (La Barbera & Ajzen, 2020b). A structural equation model of voting intentions showed an excellent fit to the data, both for the whole sample and for subsamples of young vs. old participants. Perceived behavioral control, mainly determined by participants’ beliefs about the difficulties of exerting direct democratic control through citizenship and voting, had a significant effect on intentions to vote in favor of EU integration across age groups. In addition, older people’s intentions were also affected by their attitude towards EU integration, based primarily on their beliefs about losing national identity.

## Concluding Remarks

The theory of planned behavior continues to offer a useful framework for research in the social and behavioral sciences. The studies reported in this special issue illustrate the ongoing interest in using the TPB to explain and predict behavior in various domains. At the same time, they also show that the theory is a work in progress as investigators continue to explore the intricacies of the structural model like moderating effects of perceived behavioral control and to propose additional factors to account for the complexity of human behavior.
